# Identification of lncRNA–miRNA–mRNA regulatory network associated with primary open angle glaucoma

**DOI:** 10.1186/s12886-020-01365-5

**Published:** 2020-03-16

**Authors:** Minwen Zhou, Bing Lu, Wei Tan, Mingshui Fu

**Affiliations:** 1 Department of Ophthalmology, Shanghai General Hospital (Shanghai First People’s Hospital), Shanghai Jiao Tong University School of Medicine, Shanghai, China; 2 National Clinical Research Center for Eye Diseases, Shanghai, China; 3 Shanghai Key Laboratory of Fundus Diseases, Shanghai, China; 4 The Department of Ophthalmology, The Third Affiliated Hospital of Zunyi Medical University, 98 Feng huang Road, Zunyi, China

**Keywords:** Primary open angle glaucoma, Long noncoding RNA, lncRNA-miRNA-mRNA regulatory network

## Abstract

**Background:**

Primary open angle glaucoma (POAG) is a multifactorial disorder characterized by a progressive permanent degeneration of retinal ganglion cell (RGCs) death. An increasing number of studies have suggested that long noncoding RNAs (lncRNAs) have the ability to regulate gene expression; however, thus far, the mechanisms and functions of lncRNAs in the development of POAG are still unclear.

**Methods:**

Using the data from Gene Expression Omnibus (GEO), differentially expressed lncRNAs and differentially expressed mRNAs between POAG patients and controls were identified. Then, the lncRNA–miRNA–mRNA competing endogenous RNA (ceRNA) network was constructed, and the key lncRNAs in POAG were identified. A Gene Ontology (GO) analysis and a Kyoto Encyclopedia of Genes and Genomes (KEGG) pathway analysis were performed to assess the enriched biological functions of mRNA in the ceRNA network.

**Results:**

During this study, a POAG-related ceRNA network with 37 miRNA nodes, 248 lncRNA nodes, 178 mRNA nodes, and 1985 edges was constructed. In addition, four lncRNAs (DNAJC27-AS1, AF121898, OIP5-AS1, and SNX29P2) were established as hub RNAs in this ceRNA network. The functional assay showed that 18 GO terms and 17 pathways were enriched.

**Conclusion:**

This study provides novel insights into the lncRNA-related ceRNA network in POAG, and the four lncRNAs were identified in the development of POAG.

## Background

Primary open angle glaucoma (POAG), a frequent type of glaucoma worldwide, is a complex characterized by the progressive permanent degeneration of retinal ganglion cell (RGCs) death and distinctive visual field loss [1, 2]. Regarding disease etiology, multiple risk factors, such as age [3, 4], elevated intraocular pressure (IOP) [5, 6], family history [7], and ethnic background [8], have been established in connection with the risk of POAG. In addition to these risk factors, genetics has also been shown to play a key role in the pathogenesis of POAG [9–11]. Nevertheless, the actual molecular mechanisms are still poorly understood.

A non-coding RNA (ncRNA) is an RNA molecule that is not translated into a protein. The ncRNA molecule has been found to be involved in master regulators in various biological pathologic processes [12, 13]. Long noncoding RNA (lncRNA) is a typical ncRNA with a non-protein-coding function exceeding 200 nucleotides. LncRNA has the ability to regulate gene expression by participating in regulating the transcription and translation of genes [14, 15]. Recently, emerging evidence has suggested the critical role of lncRNAs in the occurrence of POAG [9, 10, 16]; however, thus far, the mechanisms and functions of most lncRNAs remain incomprehensible with only a small portion being well-annotated.

Recently, it has been suggested that lncRNAs are emerging as competing endogenous RNAs (ceRNAs) to communicate with messenger RNA (mRNA) through competitive microRNA (miRNA). Ordinarily, miRNAs can induce target gene degradation or can inhibit mRNA translation [17]; however, lncRNA can share miRNA response elements (MREs) with mRNA and can then alleviate the inhibition of the miRNA-mediated target gene [17]. Thus, it is a competent method used to understand the complex functions of lncRNA by understanding its relationship with microRNAs and mRNAs due to the annotated functions of miRNAs and mRNAs. Thus, bioinformatic methods were carried out to analyze the gene expression profile in data sets obtained from the Gene Expression Omnibus (GEO) for the aim to further determine an innovative regulatory mechanism based on the lncRNA–miRNA–mRNA ceRNA theory in the development of POAG.

## Methods

### Sample

This bioinformatics analysis was conducted based on data that have been made publicly available; ethical approval was obtained in the original study. Written informed consent was obtained from each participant in the original study.

The information of patients was also extracted from the original study as follows. Samples for experimental groups were obtained from POAG patients who did not receive any glaucoma medication 4 months before surgery and had uncontrolled IOP. The control group for AH profiling consisted of age- and sex-matched age-related cataract patients who were candidates for cataract surgery. The mean age in the control group is 63.2 ± 3.5 years and in the POAG group it is 61.0 ± 2.9 years. The IOP were 18.8 ± 0.7 and 27.8 ± 2.1 mmHg in the control and POAG groups, respectively. The proportion of males was 48.8 and 44.2% in the control and POAG groups, respectively. Aqueous humor (approximately 100 mL) was carefully collected from patients who underwent surgery by paracentesis of the anterior chamber, using a 27-gauge needle inserted through the peripheral cornea under a microscope. During paracentesis, the needle was kept away from touching the iris and lens. All samples were immediately cooled at − 80 °C and protected from light in a dry place until they were measured.

### Expression profiles of lncRNAs and mRNAs

Using the Agilent-079487 Arraystar Human LncRNA microarray platform (GPL21827), mRNA and lncRNA expression data of GSE101727 were obtained from the publicly available GEO database (http://www.ncbi.nlm.nih.gov/geo/). The database consisted of the disease-related extracellular lncRNAs and mRNAs in the aqueous humor (AH) of individual POAG patients and the AH of individual cataracts as the control.

### Probe re-annotation

The raw data of GSE101727 only supports the sequence data format and not the gene symbol. Thus, using the software of perl (https://www.perl.org/; version 5.30.0), the sequence data format was converted to the FASTA format first. The human genome and related annotation file were obtained from the GENCODE database (https://www.gencodegenes.org). Then, the sequence alignment methods were used via NCBI-blast software (version 2.7.1). Subsequently, the probeMatrix file was converted to the gene symbol matrix file, and genes were classified as protein-coded RNA and lncRNA using the perl software.

### Differential analysis of RNAs

To establish differentially expressed lncRNA and mRNA, the limma package of the R Software for statistical analysis was used. This R package was also used to calculate the log_2_ fold change (log_2_ FC) > 1 and the false discovery rate (FDR). An adjusted *P* < 0.05 was used as the standard. The limma and heatmap packages were used to draw volcano plots and heatmaps, respectively.

### Identification of potential ceRNA interactions

Both mRNAs and lncRNAs that were negatively correlated with certain common miRNAs were defined as candidate ceRNA pairs. The mircode online tool (http://www.mircode.org/) was used to predict the lncRNA and miRNA interaction. The putative miRNA-mRNA interactions were collected from miRDB (http://mirdb.org/), TargetScan (http://www.targetscan.org/vert_72/), and miRTarBase (http://mirtarbase.mbc.nctu.edu.tw/php/index.php) databases. Only miRNA-mRNA interactions predicted by all three databases were included. Thus, all the possible miRNA targets were predicted using the perl software tool.

### Construction of the lncRNA-mediated ceRNA network

The lncRNA-mediated ceRNA network was constructed on the basis that ceRNA can bind to miRNA through MREs. This ceRNA network was visualized using Cytoscape software (Version 3.6.0) [18]. In this network, nodes and edges represented biological data in a direct manner in which each node represented a biological molecule, and the edges represented interactions between nodes [19]. LncRNAs, mRNAs, and miRNAs in the ceRNA network were presented as blue diamonds, green ellipses, and red triangles, respectively. In addition, the topological features of this ceRNA network was calculated by a built-in NetworkAnalyzer tool in Cytoscape software, including betweenness, network degree, and closeness centrality [20]. These topological parameters are standard measures of centrality in a network. Betweenness centrality was calculated as the number of shortest paths between all pairs of nodes in the network that passed through the node. The degree centrality was calculated as the number of edges linked to a node. The closeness centrality of a node was the shortest path between a node and other nodes [20]. To compare the differences in the degree, closeness, and betweenness centrality among lncRNAs, miRNAs, and mRNAs, the Kruskal–Wallis test was used.

### Gene ontology and KEGG pathway analysis

For the Gene Ontology (GO) and the Kyoto Encyclopedia of Genes and Genomes (KEGG) pathway-enrichment analysis, the clusterProfilerGO and clusterProfilerKEGG packages of the R Software were used. *P* < 0.05 was used as the cutoff criterion.

## Results

### Data set acquisition and identification of differentially expressed RNAs

**Fig. 1:**
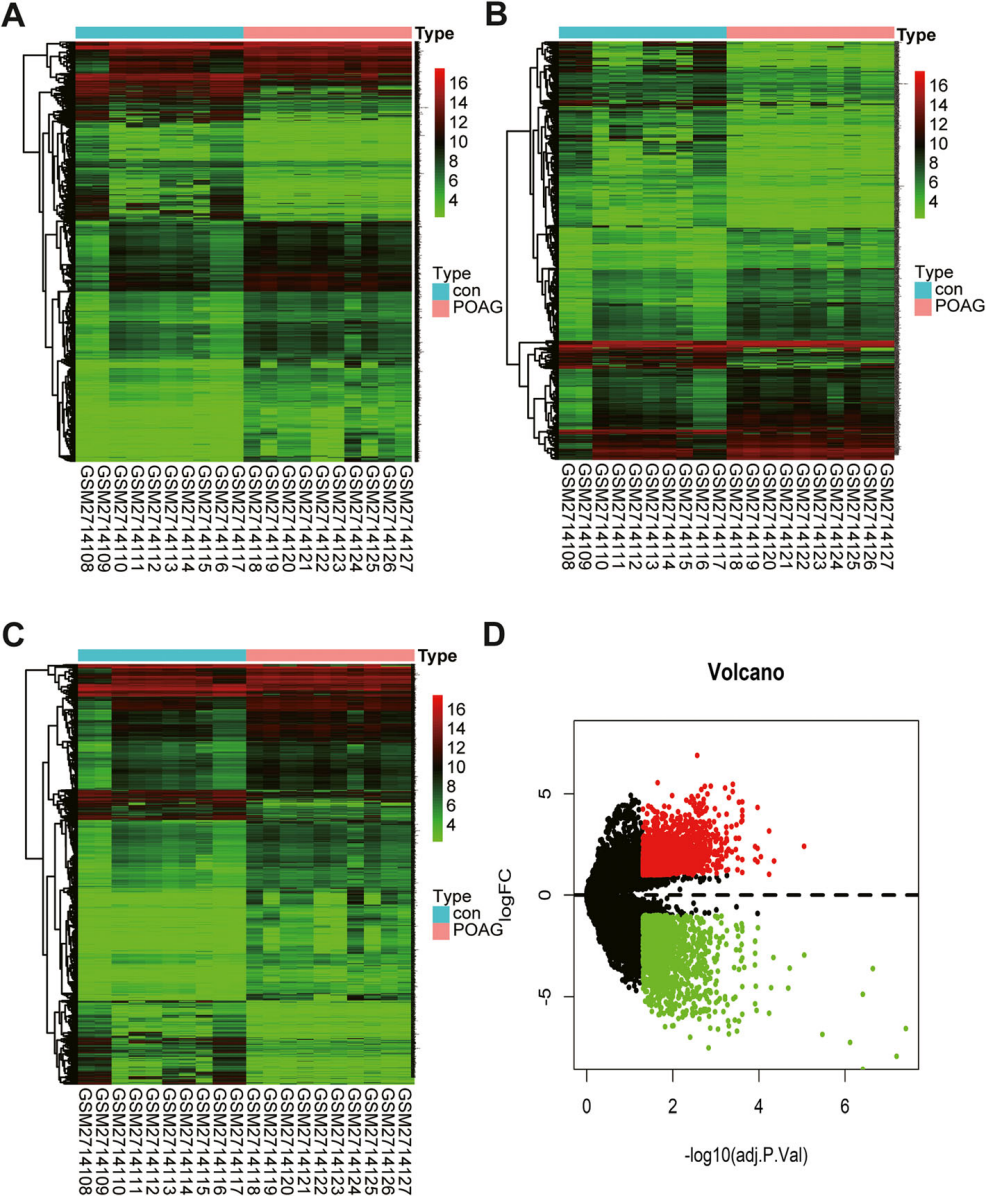
The differentially expressed RNAs in POAG. log_2_FC > 1, false discovery rate (FDR) < 0.05. **a** Heatmap plots of differentially expressed RNAs. **b** Heatmap plots of differentially expressed lncRNAs. **c** Heatmap plots of differentially expressed mRNAs. The horizontal axis represents samples. The vertical axis represents RNAs. **d** Volcano plot of differentially expressed RNAs in POAG

From the GEO database, 10 AH samples from POAG patients and 10 AH samples from cataract patients were collected. After selecting the pre-treated data based on the adjusted *P* < 0.05 and log_2_ FC > 1 change, between the 10 patients’ samples and the 10 control samples, a total of 4130 differentially expressed RNAs were identified. Among them, 1041 were lncRNA (508 up- and 533 down-regulated), and 3089 were mRNA (2135 up- and 954 down-regulated). Volcano plots of all differentially expressed genes were generated (Fig. 1). The heat maps of lncRNAs, mRNAs, and all RNAs showed the differences between POAG patients and the control group (Fig. 1).

### ceRNA network construction and analysis

**Fig. 2:**
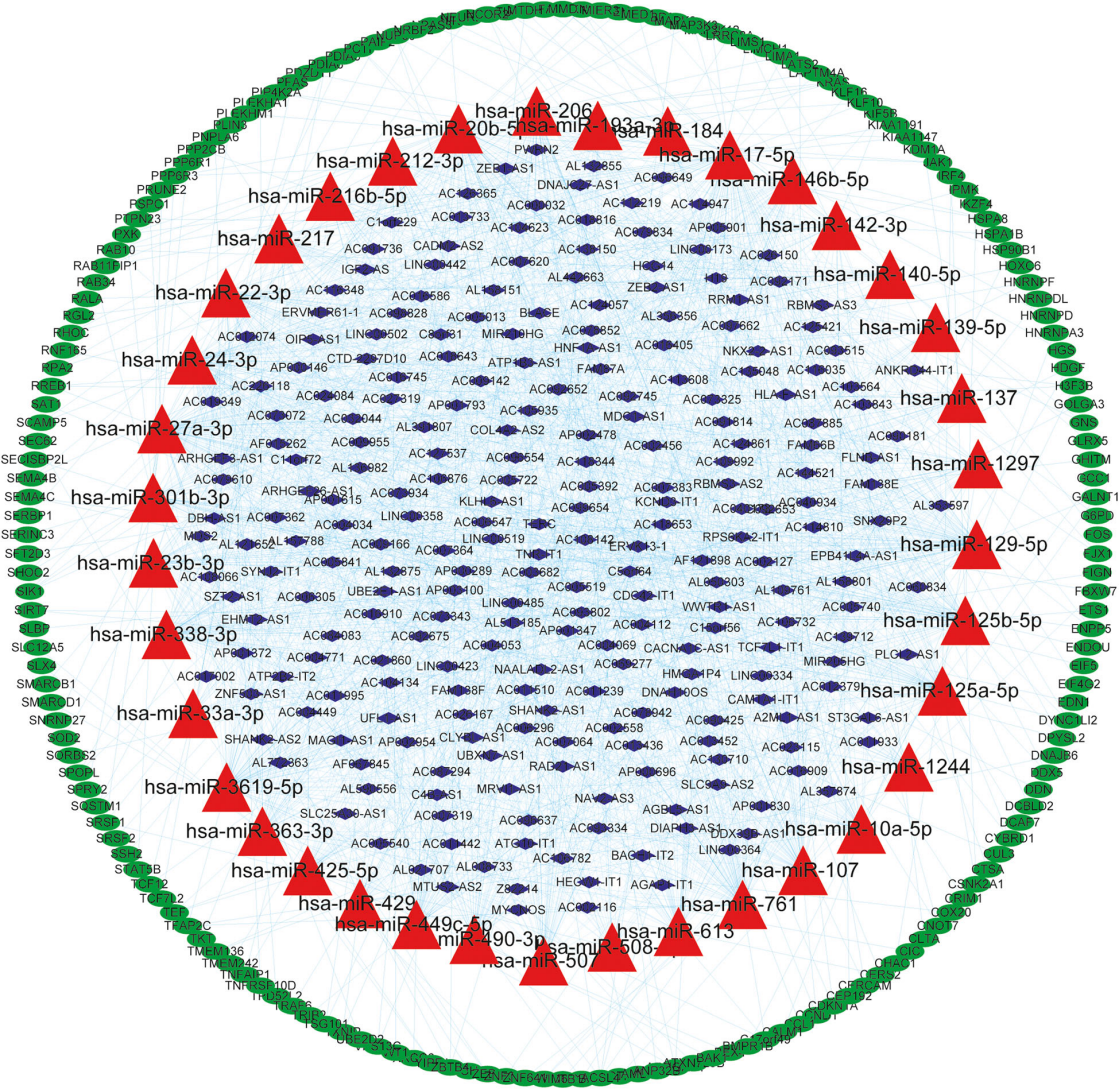
Overview of the lncRNA–miRNA–mRNA ceRNA network related with POAG. The blue diamonds, red triangles, and green ellipses nodes represented lncRNAs, miRNAs and mRNAs respectively. Blue lines represented interactions between the RNAs

**Fig. 3:**
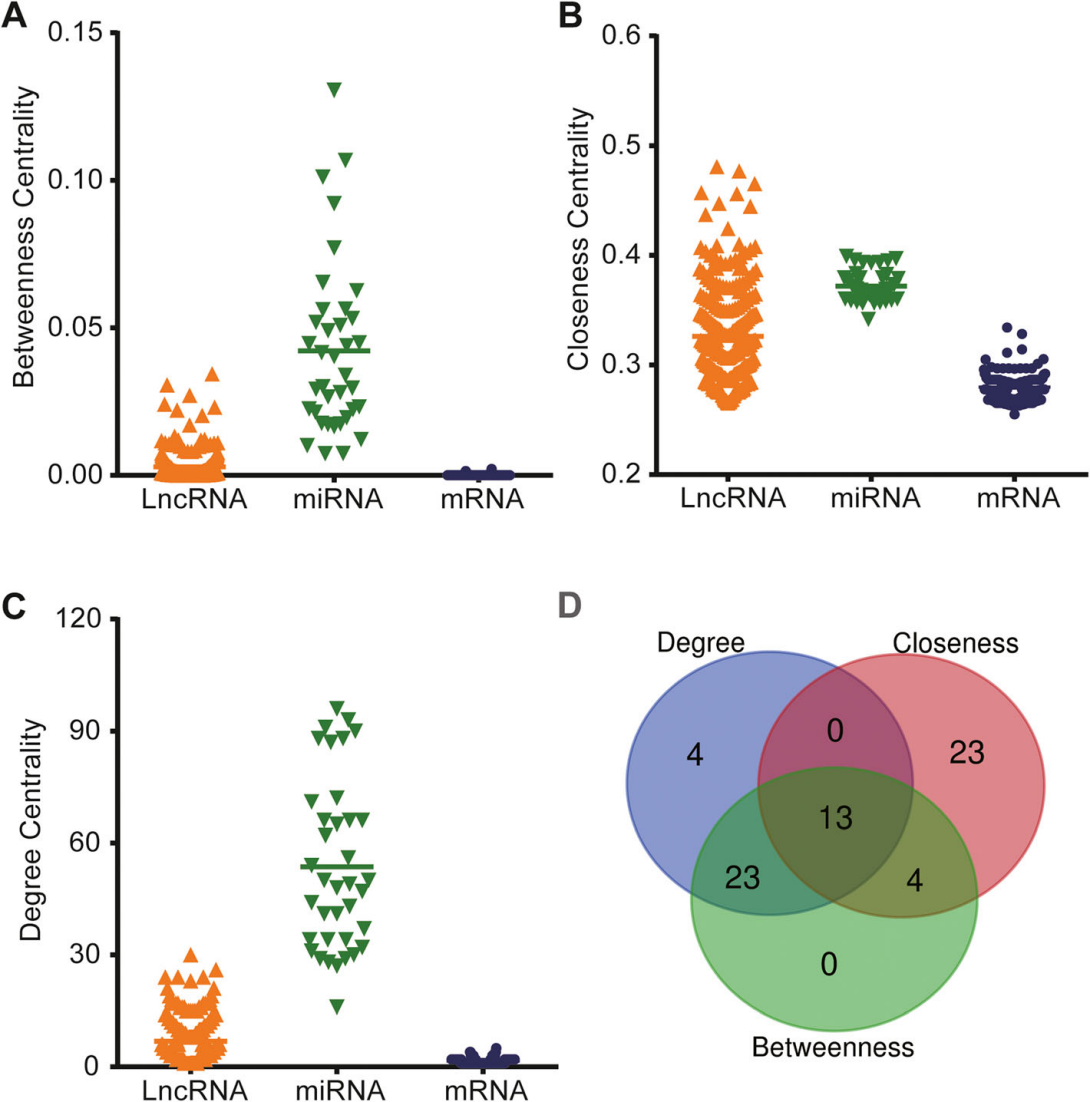
The difference in the betweenness, closeness, and degree centrality among lncRNAs, miRNAs, and mRNAs. **a** The lncRNA nodes had a significantly higher betweenness centrality than mRNA nodes in the network. **b** The lncRNA nodes had a higher closeness centrality than mRNA nodes in the network. **c** The lncRNA nodes had a higher degree centrality than mRNA nodes in the network. **d** The Venn diagram showed the overlap of top 40 genes with topological features in each dimension

The differentially expressed lncRNAs established were selected using the miRcode online tool, and the potential predicted target miRNAs were compared. Then, the relationships between differentially expressed mRNAs and miRNAs were also evaluated by the miRTarBase, MiRDB, and Targetscan databases. Finally, 248 POAG-specific lncRNAs that putatively targeted 37 POAG-specific miRNAs and the comparisons of 37 POAG-specific miRNAs and 178 POAG -specific mRNAs were involved in the ceRNA network. As displayed in Fig. 2, 37 miRNA nodes, 248 lncRNA nodes, 178 mRNA nodes, and 1985 edges comprised the lncRNA–miRNA–mRNA network. The topological features of this ceRNA network were assessed by a built-in NetworkAnalyzer tool in Cytoscape software, including betweenness, network degree, and closeness centrality. Generally, the nodes in the ceRNA network with a higher degree, closeness, and betweenness centrality demonstrated a higher possibility of hub nodes in the ceRNA network. A Venn diagram for the overlapping top 40 genes with topological features in each dimension was created (Table 1, Fig. 3d). Finally, nine miRNAs (hsa-miR-20b-5p, hsa-miR-761, hsa-miR-17-5p, hsa-miR-338-3p, hsa-miR-24-3p, hsa-miR-125b-5p, hsa-miR-3619-5p, hsa-miR-129-5p, and hsa-miR-27a) and four lncRNAs (DNAJC27-AS1, AF121898, OIP5-AS1, and SNX29P2) were established as hub RNAs in the ceRNA network. When comparing the differences in the degree, closeness, and betweenness centrality among lncRNAs, miRNAs, and mRNAs, the result showed that lncRNAs and miRNAs had a higher degree, closeness, and betweenness centrality than mRNAs (Fig. 3), indicating that lncRNAs and miRNAs tended to be pivotal to the risk of POAG.

**Table 1:** Hub RNAs in the ceRNA network

RNAs	Closeness	Degree	Betweenness
OIP5-AS1	0.48074922	30	0.0343011
SNX29P2	0.47678019	26	0.0305805
DNAJC27-AS1	0.45607108	24	0.0230366
AF121898	0.44724105	24	0.0220339
hsa-miR-17-5p	0.39896373	96	0.1305271
hsa-miR-27a-3p	0.39690722	93	0.1068025
hsa-miR-3619-5p	0.39554795	91	0.0561762
hsa-miR-761	0.39487179	90	0.0518472
hsa-miR-24-3p	0.39352641	88	0.0769798
hsa-miR-129-5p	0.39352641	88	0.1011164
hsa-miR-20b-5p	0.39285714	87	0.0920732
hsa-miR-338-3p	0.38308458	72	0.0416175
hsa-miR-125b-5p	0.38245033	71	0.0625927

### Functional annotation of the ceRNA network

**Fig. 4:**
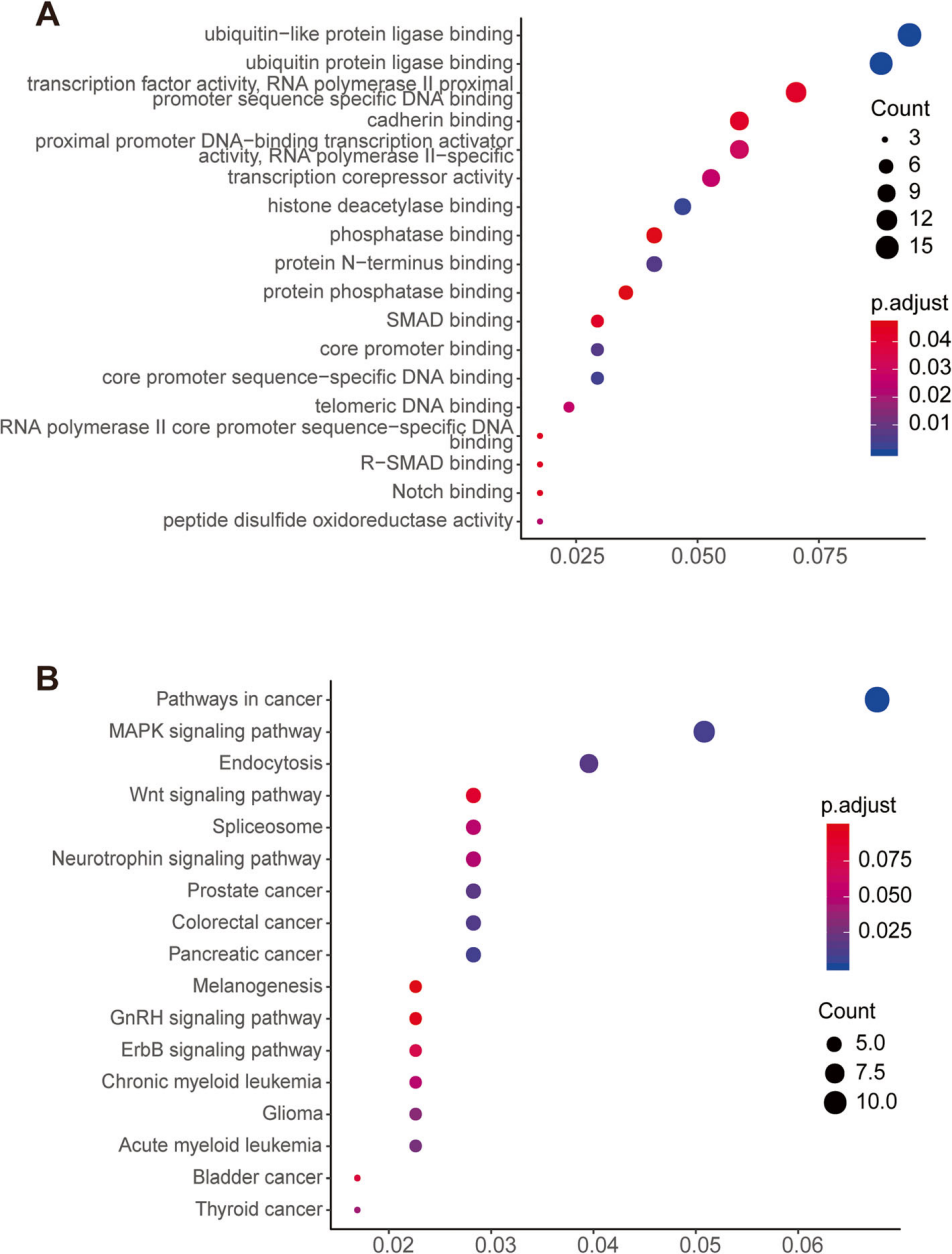
Gene ontology (**a**) and KEGG pathway (**b**) enriched analysis of the mRNAs in the ceRNA network. Y-axis label represents terms name, and X-axis label represents gene ratio which is defined as the percentage of target genes per term

To achieve a more thorough understanding of the mRNAs’ function in the ceRNA network, a GO and a KEGG analysis were performed using R software. The functional assay showed that 18 GO terms and 17 pathways were enriched. For the GO analysis, POAG was significantly enriched in ubiquitin-like protein ligase binding, ubiquitin protein ligase binding, transcription factor activity, etc. The KEGG-enriched analysis results indicated that major pathways, including the mitogen-activated protein kinase (MAPK) signaling pathway, endocytosis pathway, and Wnt signaling pathway, were involved in these mRNAs. The results of the GO and KEGG enriched analyses are displayed in Fig. 4.

## Discussion

POAG is a multifactorial disorder with various etiologies that is estimated to occur in about 52 million patients in 2020 around the world [8]. During the last few years, considerable efforts have been made to investigate the molecular mechanisms of POAG [21, 22]; however, most previous studies have mainly focused on protein-coding genes or miRNAs but not on lncRNAs [21–23].

It has been reported that more than 10,000 lncRNAs are produced by human genomes; however, to date, little information related to lncRNAs, especially their function, have been established [24, 25]. Recently, lncRNA has been found to be involved in some important regulatory processes, including transcriptional interference, transcriptional activation, and chromatin modification, and it may serve as a biomarker for various diseases, such as lung cancer [26], colorectal cancer [27], diabetes mellitus [28], and liver fibrosis [29]. Nevertheless, few studies have reported the role of lncRNA in the pathogenesis of POAG. Thus, the identification of lncRNA in the pathogenesis of POAG is necessary.

The newly emerged ceRNA hypothesis has been suggested as an innovative post-transcriptional regulatory mechanism of gene expression. Under this ceRNA network, the lncRNAs and mRNAs are connected by their common target miRNAs [30]. In recent years, several studies have explored the underlying molecular mechanisms based on the ceRNA network in some diseases, such as breast cancer [31], ischemic stroke [32], and rheumatoid arthritis [33]. To identify lncRNAs significantly associated with POAG, in this study, the mRNA and lncRNA expression profiles of POAG patients were first used and combined with miRNA-target interactions to create a ceRNA network and to investigate the potential implications of these lncRNAs in the development of POAG.

In this study, four lncRNAs (DNAJC27-AS1, AF121898, OIP5-AS1, and SNX29P2) served as the hub nodes finally. In addition, the GO and KEGG pathway analyses were used to assess enriched biological functions. The differentially expressed mRNA in the lncRNA–miRNA–mRNA ceRNA network-related GO analysis showed that ubiquitin-like protein ligase, ubiquitin protein ligase, and others could play an important role in the development of POAG. The pathway analysis further revealed that 17 unique pathways were enriched, including the MAPK signaling pathway, endocytosis pathway, and Wnt signaling pathway. In fact, an increasing amount of experimental evidence has indicated that these enriched pathways have always been involved in glaucoma. For example, Beit-Yannai et al. [34] found that AH in a rat model of induced elevated IOP expressed several signaling members of the MAPK family; they suggested that MAPKs present in the aqueous humor are a novel signal involved in glaucoma pathology. In addition, another study showed that the MAPK signal pathway participates in protecting human trabecular meshwork cells from pressure-induced apoptosis [35]. Webber et al. [36] reported that Wnt signaling pathways play important roles in the regulation of TM homeostasis and IOP [37]. Thus, the enrichment results could suggest that the lncRNA–miRNA–mRNA ceRNA network plays an important role, by way of these pathways, in the development of POAG.

Hub nodes, which have been examined in some studies and are characterized by their high degree of connectivity to other nodes in the ceRNA network, can be used as topological properties of the ceRNA network to assess the significance of genes [38, 39]. In the present study, four lncRNAs (DNAJC27-AS1, AF121898, OIP5-AS1, and SNX29P2) were observed to be topological hub nodes whose betweenness, network degree, and closeness centrality were significantly higher in comparison with other lncRNAs. Thus far, among these hub lncRNAs, DNAJC27-AS1, AF121898, and SNX29P2 have not been reported in any study. OIP5-AS1 is an antisense lncRNA that has been reported to play a critical role in various disorders, including oral squamous cell carcinoma [40], gastric cancer [41], cardiovascular disease [42], and multiple sclerosis [43]. Li and colleague’s study revealed that OIP5-AS1 could promote the progression of oral squamous cell carcinoma by regulating the miR-338-3p/NRP1 axis [40]. Another study has also indicated an aggressive role of ceRNA to drive migration, invasion, and proliferation of human hemangioma endothelial cells via regulating the miR-195-5p/NOB1 axis [44]. In the present study, it was observed that OIP5-AS1 displayed low-expression, which could compete with miRNAs (hsa-miR-17-5p, hsa-miR-20b-5p, hsa-miR-761, hsa-miR-3619-5p, hsa-miR-24-3p, hsa-miR-27a, hsa-miR-338-3p, and hsa-miR-129-5p) to regulate target gene expression. These miRNAs interacted with OIP5-AS1 and have been known to be involved in glaucoma. For example, Zhao’s study has confirmed that miR-27a (a target miRNA of OIP5-AS1) has protective impacts on H2O2-injured human trabecular meshwork cells, which comprise a common glaucoma cell model [45]. Another miRNA (miR-17-5p, a target miRNA of OIP5-AS1) has been found to have the function of regulating the proliferation and apoptosis of human trabecular meshwork cells in response to oxidative stress [46]. Up to now, the research on OIP5-AS1 in glaucoma is still blank. The findings of our study show that OIP5-AS1 may be related to the development of glaucoma via lncRNA–miRNA–mRNA ceRNA network analysis. Therefore, the OIP5-AS1/miRNA/mRNA axis may become a hot issue for the study of glaucoma in the future.

Although the findings of this study have important clinical significance, the limitations should be discussed. First, the conclusion of this study based on the GEO database should be verified by other experimental evidence. Second, the precise cellular sources and mechanisms underlying hub genes, such as OIP5-AS1, DNAJC27-AS1, AF121898, and SNX29P2, in relation to POAG should be further investigated. Third, from the GEO database, 10 AH samples from POAG patients and 10 AH samples from cataract patients is a relatively small sample; this is the major drawback when performing genetic studies of association. Thus, the conclusion and the results of this study should be interpreted with caution.

## Conclusions

In summary, during this study, a POAG-related lncRNA–miRNA–mRNA ceRNA network was constructed, and hub lncRNAs, such as OIP5-AS1, DNAJC27-AS1, AF121898, and SNX29P2, were identified in the development of POAG, which provided novel insights into exploring the underlying mechanism of POAG. Further experimental studies should be performed to elucidate the molecular mechanisms underlying the lncRNA function in POAG.

## Data Availability

The datasets used and/or analyzed during the current study are available from the corresponding author on reasonable request.

## Abbreviations

AH: Aqueous humor
ceRNA: Competing endogenous RNA
FDR: False discovery rate
GEO: Gene Expression Omnibus
GO: Gene Ontology
IOP: Intraocular pressure
KEGG: Kyoto Encyclopedia of Genes and Genomes
lncRNA: Long noncoding RNA
log_2_ FC: Log_2_ fold change
miRNA: MicroRNA
MRE: MiRNA response element
mRNA: Messenger RNA
ncRNA: Non-coding RNA
POAG: Primary open angle glaucoma
RGC: Retinal ganglion cell
